# (2*Z*)-*N*-(4-Meth­oxy­phen­yl)-2-(4-meth­oxy­phenyl­imino)-2*H*-1,4-benzoxazin-3-amine

**DOI:** 10.1107/S1600536810050294

**Published:** 2010-12-08

**Authors:** Morteza Mehrdad, Mohammad Ghanbari, Khosrow Jadidi, Amir Salemi, Hamid Reza Khavasi

**Affiliations:** a Department of Environmental Pollution, Environmental Sciences Research Institute, Shahid Beheshti University, G.C., Evin, Tehran 1983963113, Iran; b Department of Chemistry, Shahid Beheshti University, G. C., Evin, Tehran 1983963113, Iran

## Abstract

In the crystal structure of the title compound, C_22_H_19_N_3_O_3_, inter­molecular C—H⋯O hydrogen bonds link the mol­ecules into a zigzag chain parallel to the face diagonal of the *ac* plane. The meth­oxy phenyl rings make a dihdral angle of 32.38 (7)° and form dihedral angles of 0.66 (8) and 24.17 (7)° with the fused benzooxazine ring system.

## Related literature

For the Baeyer–Villiger oxidation of 1-alkyl-3-aryl­imino-2-indolinone with *m*-chloro­perbenzoic acid to afford 1-alkyl-4-(aryl­imino)-1*H* benzo[*d*][1,3]oxazin-2(4*H*)-one, see: Mehrdad *et al.* (2011 ▶); Azizian *et al.* (2000 ▶); Jadidi *et al.* (2008 ▶). For a related structure, see: Asgari *et al.* (2011 ▶).
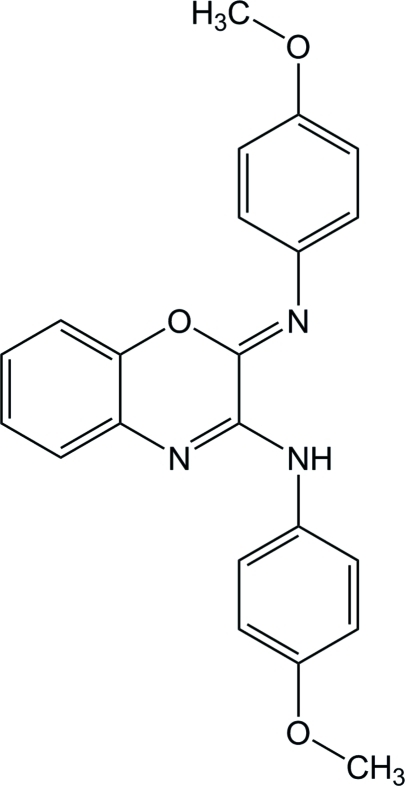


## Experimental

### 

#### Crystal data

C_22_H_19_N_3_O_3_
*M* *_r_* = 373.40
Monoclinic, 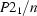

*a* = 14.4225 (14) Å
*b* = 8.0836 (5) Å
*c* = 16.2749 (14) Å
β = 107.263 (7)°
*V* = 1811.9 (3) Å^3^
*Z* = 4
Mo *K*α radiation
μ = 0.09 mm^−1^
*T* = 298 K
0.60 × 0.13 × 0.04 mm

#### Data collection

Stoe IPDS II diffractometer
21467 measured reflections
4893 independent reflections
3190 reflections with *I* > 2σ(*I*)
*R*_int_ = 0.111

#### Refinement

*R*[*F* ^2^ > 2σ(*F* ^2^)] = 0.083
*wR*(*F* ^2^) = 0.195
*S* = 1.15
4893 reflections
253 parameters
H-atom parameters constrained
Δρ_max_ = 0.24 e Å^−3^
Δρ_min_ = −0.28 e Å^−3^

### 

Data collection: *X-AREA* (Stoe & Cie, 2005 ▶); cell refinement: *X-AREA*; data reduction: *X-AREA*; program(s) used to solve structure: *SHELXS97* (Sheldrick, 2008 ▶); program(s) used to refine structure: *SHELXL97* (Sheldrick, 2008 ▶); molecular graphics: *ORTEP-3 for Windows* (Farrugia, 1997 ▶); software used to prepare material for publication: *WinGX* (Farrugia, 1999 ▶).

## Supplementary Material

Crystal structure: contains datablocks I, global. DOI: 10.1107/S1600536810050294/bt5425sup1.cif

Structure factors: contains datablocks I. DOI: 10.1107/S1600536810050294/bt5425Isup2.hkl

Additional supplementary materials:  crystallographic information; 3D view; checkCIF report

## Figures and Tables

**Table 1:** Hydrogen-bond geometry (Å, °)

*D*—H⋯*A*	*D*—H	H⋯*A*	*D*⋯*A*	*D*—H⋯*A*
C7—H7⋯O3^i^	0.93	2.59	3.423 (3)	149

Symmetry code: (i) 
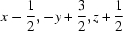
.

**Fig. 1.**
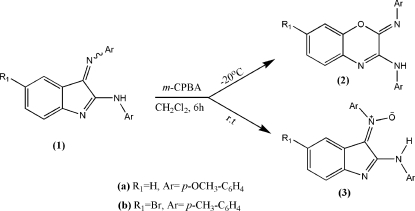
Reaction scheme.

**Fig. 2.**
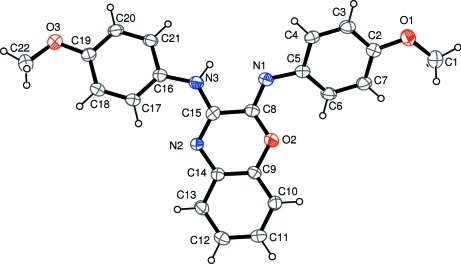
The molecular structure of the title molecule, with the atom-numbering scheme. Displacement ellipsoids are drawn at the 30% probability level.

**Fig. 3.**
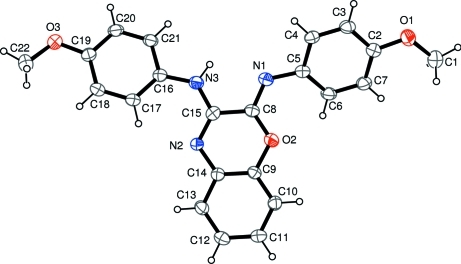
Unit-cell packing diagram for (I).

## supplementary crystallographic information

### Comment

Recently, we reported a Baeyer–Villiger oxidation of 1-alkyl-3-arylimino-2-indolinone with *m*-chloroperbenzoic acid to afford 1-alkyl-4-(arylimino)-1H benzo[*d*][1,3]oxazin-2(4*H*)-one (Azizian *et al.*, 2000; Jadidi *et al.*, 2008). As a continuation of this work, 2-arylimino-*N*-aryl-2*H*-benzo[*b*][1,4]oxazin-3-amines (2) or *N*-aryl-*N*-(2-arylamino-3*H*-indol-3-ylidene)amine N-oxides (3) were obtained in two different temperatures by Baeyer-Villiger oxidation reaction (Fig. 1) of *N*-aryl-3-(arylimino)-3*H*-indol-2-amines (1) (Mehrdad *et al.*, 2011). In this paper, we report the structure of (2*Z*)-2-(4-methoxyphenylimino)-*N*-(4-methoxyphenyl)- 2*H*-benzo[*b*][1,4]oxazin-3-amine (2a). The molecular structure of the title compound is shown in Fig. 2.

The methoxy phenyl rings, A (C2—C7) and B (C16—C21) and benzooxazine ring C (C9—C14/C8/O2/N2/C15) enclose the  dihedral angles: A/B = 32.38 (7)°, A/C = 10.66 (8)° and B/C = 24.17 (7)°. Intermolecular C—H···O interactions (Table 1)   stabilize the crystal structure.

### Experimental

The solution of *N*-Aryl-3-(Arylimino)-3*H*-indol-2-amine (1a) (1.0 mmol) in 25 ml CH_2_Cl_2_ was cooled to 253K. Then, *m*-CPBA (1.5 mmol) dissolved in 25 ml CH_2_Cl_2_ was added dropwise to the stirred solution of (1a). After stirring for 6 h at 253K, product (2a) was formed (monitoring by TLC). The crude product was poured into water and extracted with CH_2_Cl_2_ (60 ml). The organic layer was dried over Na_2_SO_4_, and evaporation of the solvent afforded the crude product (2a), which was purified on silica gel by column chromatography using 90:10 n-hexane:ethyl acetate as eluent to afford (2a) as a light yellow solid (90%); m.p. = 169–171°C (Mehrdad *et al.*, 2011).

### Refinement

All H atoms were positioned geometrically, with N—H=0.86 Å, C_methyl_—H=0.96Å and C_aromatic_—H=0.93Å and constrained to ride on their parent atoms, with *U*_iso_(H)=1.2*U*_eq_(C,N).

### Crystal data

**Table:**

C_22_H_19_N_3_O_3_	*F*(000) = 784
*M**_r_* = 373.40	*D*_x_ = 1.369 Mg m^−^^3^
Monoclinic, *P*2_1_/*n*	Mo *K*α radiation, λ = 0.71073 Å
Hall symbol: -P 2yn	Cell parameters from 21467 reflections
*a* = 14.4225 (14) Å	θ = 1.7–29.3°
*b* = 8.0836 (5) Å	µ = 0.09 mm^−^^1^
*c* = 16.2749 (14) Å	*T* = 298 K
β = 107.263 (7)°	Needle, yellow
*V* = 1811.9 (3) Å^3^	0.60 × 0.13 × 0.04 mm
*Z* = 4	

### Data collection

**Table:**

Stoe IPDS II diffractometer	3190 reflections with *I* > 2σ(*I*)
Radiation source: fine-focus sealed tube	*R*_int_ = 0.111
graphite	θ_max_ = 29.3°, θ_min_ = 1.7°
Detector resolution: 0.15 mm pixels mm^-1^	*h* = −18→19
rotation method scans	*k* = −10→11
21467 measured reflections	*l* = −22→22
4893 independent reflections	

### Refinement

**Table:**

Refinement on *F*^2^	Primary atom site location: structure-invariant direct methods
Least-squares matrix: full	Secondary atom site location: difference Fourier map
*R*[*F*^2^ > 2σ(*F*^2^)] = 0.083	Hydrogen site location: inferred from neighbouring sites
*wR*(*F*^2^) = 0.195	H-atom parameters constrained
*S* = 1.15	*w* = 1/[σ^2^(*F*_o_^2^) + (0.0578*P*)^2^ + 0.8502*P*] where *P* = (*F*_o_^2^ + 2*F*_c_^2^)/3
4893 reflections	(Δ/σ)_max_ = 0.002
253 parameters	Δρ_max_ = 0.24 e Å^−^^3^
0 restraints	Δρ_min_ = −0.28 e Å^−^^3^

### Special details

**Table:**

Geometry. All e.s.d.'s (except the e.s.d. in the dihedral angle between two l.s. planes) are estimated using the full covariance matrix. The cell e.s.d.'s are taken into account individually in the estimation of e.s.d.'s in distances, angles and torsion angles; correlations between e.s.d.'s in cell parameters are only used when they are defined by crystal symmetry. An approximate (isotropic) treatment of cell e.s.d.'s is used for estimating e.s.d.'s involving l.s. planes.
Refinement. Refinement of *F*^2^ against ALL reflections. The weighted *R*-factor *wR* and goodness of fit *S* are based on *F*^2^, conventional *R*-factors *R* are based on *F*, with *F* set to zero for negative *F*^2^. The threshold expression of *F*^2^ > σ(*F*^2^) is used only for calculating *R*-factors(gt) *etc*. and is not relevant to the choice of reflections for refinement. *R*-factors based on *F*^2^ are statistically about twice as large as those based on *F*, and *R*- factors based on ALL data will be even larger.

### Fractional atomic coordinates and isotropic or equivalent isotropic displacement parameters (Å^2^)

**Table:**

	*x*	*y*	*z*	*U*_iso_*/*U*_eq_	
C1	−0.0070 (3)	0.2134 (5)	0.0453 (2)	0.0719 (9)	
H1A	−0.0208	0.3142	0.0707	0.086*	
H1B	0.0435	0.1542	0.0866	0.086*	
H1C	−0.0645	0.1464	0.0281	0.086*	
C2	0.1045 (2)	0.3483 (3)	−0.01638 (17)	0.0497 (6)	
C3	0.1271 (2)	0.3897 (3)	−0.09054 (17)	0.0521 (7)	
H3	0.0870	0.3551	−0.1437	0.063*	
C4	0.2088 (2)	0.4820 (3)	−0.08625 (16)	0.0476 (6)	
H4	0.2224	0.5109	−0.1368	0.057*	
C5	0.27136 (19)	0.5328 (3)	−0.00716 (16)	0.0459 (6)	
C6	0.2465 (2)	0.4957 (4)	0.06647 (17)	0.0557 (7)	
H6	0.2861	0.5319	0.1196	0.067*	
C7	0.1629 (2)	0.4047 (4)	0.06239 (18)	0.0570 (7)	
H7	0.1465	0.3822	0.1124	0.068*	
C8	0.43609 (19)	0.6338 (3)	0.05119 (16)	0.0446 (6)	
C9	0.54000 (18)	0.5791 (3)	0.18936 (16)	0.0436 (6)	
C10	0.5543 (2)	0.5004 (4)	0.26776 (18)	0.0518 (6)	
H10	0.5058	0.4342	0.2773	0.062*	
C11	0.6412 (2)	0.5210 (4)	0.33167 (17)	0.0536 (7)	
H11	0.6514	0.4685	0.3844	0.064*	
C12	0.7131 (2)	0.6199 (4)	0.31721 (18)	0.0554 (7)	
H12	0.7713	0.6340	0.3606	0.066*	
C13	0.6990 (2)	0.6979 (4)	0.23896 (17)	0.0525 (7)	
H13	0.7476	0.7644	0.2298	0.063*	
C14	0.61137 (18)	0.6767 (3)	0.17343 (15)	0.0428 (6)	
C15	0.5157 (2)	0.7295 (3)	0.03396 (16)	0.0447 (6)	
C16	0.55436 (18)	0.8817 (3)	−0.08379 (15)	0.0435 (6)	
C17	0.6539 (2)	0.9088 (4)	−0.04862 (17)	0.0536 (7)	
H17	0.6862	0.8660	0.0053	0.064*	
C18	0.7048 (2)	0.9990 (4)	−0.09332 (18)	0.0578 (7)	
H18	0.7713	1.0150	−0.0693	0.069*	
C19	0.6583 (2)	1.0656 (3)	−0.17326 (17)	0.0487 (6)	
C20	0.5592 (2)	1.0388 (4)	−0.20832 (17)	0.0548 (7)	
H20	0.5268	1.0827	−0.2620	0.066*	
C21	0.5087 (2)	0.9484 (4)	−0.16457 (16)	0.0505 (6)	
H21	0.4425	0.9312	−0.1894	0.061*	
C22	0.8046 (2)	1.1618 (5)	−0.1983 (2)	0.0777 (10)	
H22A	0.8289	1.0517	−0.1997	0.093*	
H22B	0.8296	1.2060	−0.1412	0.093*	
H22C	0.8250	1.2305	−0.2379	0.093*	
N1	0.35461 (16)	0.6221 (3)	−0.01063 (14)	0.0499 (5)	
N2	0.59911 (15)	0.7521 (3)	0.09392 (12)	0.0413 (5)	
N3	0.49602 (16)	0.7909 (3)	−0.04487 (13)	0.0494 (5)	
H3A	0.4381	0.7720	−0.0774	0.059*	
O1	0.02353 (17)	0.2511 (3)	−0.02758 (14)	0.0700 (6)	
O2	0.45078 (13)	0.5596 (3)	0.12731 (12)	0.0556 (5)	
O3	0.70194 (16)	1.1580 (3)	−0.22196 (13)	0.0655 (6)	

### Atomic displacement parameters (Å^2^)

**Table:**

	*U*^11^	*U*^22^	*U*^33^	*U*^12^	*U*^13^	*U*^23^
C1	0.066 (2)	0.077 (2)	0.076 (2)	−0.0097 (17)	0.0262 (17)	0.0031 (18)
C2	0.0490 (15)	0.0481 (15)	0.0498 (14)	0.0023 (12)	0.0114 (12)	−0.0024 (12)
C3	0.0606 (17)	0.0492 (15)	0.0416 (13)	−0.0021 (13)	0.0074 (12)	−0.0070 (12)
C4	0.0567 (16)	0.0476 (15)	0.0367 (12)	0.0030 (12)	0.0111 (11)	−0.0008 (11)
C5	0.0479 (14)	0.0464 (14)	0.0405 (12)	0.0063 (11)	0.0088 (11)	−0.0015 (11)
C6	0.0488 (15)	0.075 (2)	0.0393 (13)	−0.0014 (14)	0.0072 (11)	−0.0043 (13)
C7	0.0549 (17)	0.075 (2)	0.0414 (13)	−0.0001 (15)	0.0143 (12)	0.0018 (13)
C8	0.0438 (13)	0.0448 (14)	0.0420 (13)	0.0052 (11)	0.0077 (11)	−0.0031 (11)
C9	0.0378 (13)	0.0457 (14)	0.0445 (13)	0.0027 (10)	0.0079 (10)	0.0008 (11)
C10	0.0464 (14)	0.0535 (16)	0.0554 (15)	0.0022 (12)	0.0147 (12)	0.0093 (13)
C11	0.0533 (16)	0.0587 (17)	0.0434 (13)	0.0080 (13)	0.0061 (12)	0.0054 (13)
C12	0.0479 (15)	0.0627 (18)	0.0486 (14)	−0.0022 (13)	0.0035 (12)	−0.0079 (13)
C13	0.0467 (15)	0.0584 (17)	0.0499 (15)	−0.0085 (12)	0.0104 (12)	−0.0057 (13)
C14	0.0438 (13)	0.0431 (13)	0.0414 (12)	0.0015 (11)	0.0125 (10)	−0.0020 (10)
C15	0.0531 (15)	0.0393 (13)	0.0443 (13)	0.0065 (11)	0.0186 (11)	−0.0017 (10)
C16	0.0440 (13)	0.0461 (14)	0.0400 (12)	0.0054 (11)	0.0119 (11)	−0.0026 (10)
C17	0.0440 (14)	0.0676 (18)	0.0433 (14)	0.0022 (13)	0.0036 (12)	0.0082 (13)
C18	0.0421 (14)	0.075 (2)	0.0497 (14)	−0.0037 (14)	0.0041 (12)	0.0031 (15)
C19	0.0533 (15)	0.0501 (15)	0.0423 (13)	0.0014 (12)	0.0136 (12)	−0.0018 (11)
C20	0.0530 (16)	0.0699 (19)	0.0372 (12)	0.0059 (14)	0.0068 (12)	0.0054 (13)
C21	0.0422 (13)	0.0640 (18)	0.0408 (13)	0.0028 (12)	0.0052 (11)	−0.0016 (12)
C22	0.0560 (19)	0.095 (3)	0.084 (2)	−0.0101 (18)	0.0233 (18)	0.012 (2)
N1	0.0459 (12)	0.0551 (14)	0.0447 (11)	−0.0003 (10)	0.0071 (10)	−0.0023 (10)
N2	0.0417 (11)	0.0446 (12)	0.0361 (10)	−0.0024 (9)	0.0095 (8)	−0.0002 (9)
N3	0.0446 (12)	0.0576 (14)	0.0431 (11)	0.0033 (10)	0.0087 (9)	0.0008 (10)
O1	0.0668 (14)	0.0815 (15)	0.0617 (13)	−0.0229 (12)	0.0193 (11)	−0.0067 (11)
O2	0.0446 (10)	0.0604 (12)	0.0558 (11)	−0.0025 (9)	0.0058 (9)	0.0078 (9)
O3	0.0595 (13)	0.0817 (15)	0.0544 (11)	−0.0078 (11)	0.0157 (10)	0.0101 (11)

### Geometric parameters (Å, °)

**Table:**

C1—O1	1.416 (4)	C11—H11	0.9300
C1—H1A	0.9600	C12—C13	1.381 (4)
C1—H1B	0.9600	C12—H12	0.9300
C1—H1C	0.9600	C13—C14	1.402 (4)
C2—O1	1.374 (3)	C13—H13	0.9300
C2—C3	1.382 (4)	C14—N2	1.393 (3)
C2—C7	1.386 (4)	C15—N2	1.318 (3)
C3—C4	1.378 (4)	C15—N3	1.325 (3)
C3—H3	0.9300	C16—C21	1.393 (4)
C4—C5	1.397 (3)	C16—C17	1.396 (4)
C4—H4	0.9300	C16—N3	1.401 (3)
C5—C6	1.382 (4)	C17—C18	1.385 (4)
C5—N1	1.417 (4)	C17—H17	0.9300
C6—C7	1.397 (4)	C18—C19	1.384 (4)
C6—H6	0.9300	C18—H18	0.9300
C7—H7	0.9300	C19—O3	1.370 (3)
C8—N1	1.304 (3)	C19—C20	1.390 (4)
C8—O2	1.336 (3)	C20—C21	1.371 (4)
C8—C15	1.479 (4)	C20—H20	0.9300
C9—C14	1.381 (4)	C21—H21	0.9300
C9—C10	1.385 (4)	C22—O3	1.415 (4)
C9—O2	1.389 (3)	C22—H22A	0.9600
C10—C11	1.381 (4)	C22—H22B	0.9600
C10—H10	0.9300	C22—H22C	0.9600
C11—C12	1.384 (4)	N3—H3A	0.8600
			
O1—C1—H1A	109.5	C12—C13—H13	120.1
O1—C1—H1B	109.5	C14—C13—H13	120.1
H1A—C1—H1B	109.5	C9—C14—N2	121.9 (2)
O1—C1—H1C	109.5	C9—C14—C13	118.8 (2)
H1A—C1—H1C	109.5	N2—C14—C13	119.4 (2)
H1B—C1—H1C	109.5	N2—C15—N3	123.5 (2)
O1—C2—C3	115.8 (2)	N2—C15—C8	121.4 (2)
O1—C2—C7	124.8 (3)	N3—C15—C8	115.1 (2)
C3—C2—C7	119.5 (3)	C21—C16—C17	117.8 (2)
C4—C3—C2	120.5 (2)	C21—C16—N3	116.8 (2)
C4—C3—H3	119.8	C17—C16—N3	125.4 (2)
C2—C3—H3	119.8	C18—C17—C16	120.6 (2)
C3—C4—C5	121.0 (2)	C18—C17—H17	119.7
C3—C4—H4	119.5	C16—C17—H17	119.7
C5—C4—H4	119.5	C19—C18—C17	120.9 (3)
C6—C5—C4	118.2 (3)	C19—C18—H18	119.5
C6—C5—N1	125.9 (2)	C17—C18—H18	119.5
C4—C5—N1	115.9 (2)	O3—C19—C18	125.3 (3)
C5—C6—C7	121.0 (3)	O3—C19—C20	116.1 (2)
C5—C6—H6	119.5	C18—C19—C20	118.5 (3)
C7—C6—H6	119.5	C21—C20—C19	120.7 (2)
C2—C7—C6	119.8 (3)	C21—C20—H20	119.7
C2—C7—H7	120.1	C19—C20—H20	119.7
C6—C7—H7	120.1	C20—C21—C16	121.4 (2)
N1—C8—O2	122.8 (2)	C20—C21—H21	119.3
N1—C8—C15	117.7 (2)	C16—C21—H21	119.3
O2—C8—C15	119.5 (2)	O3—C22—H22A	109.5
C14—C9—C10	121.3 (2)	O3—C22—H22B	109.5
C14—C9—O2	120.6 (2)	H22A—C22—H22B	109.5
C10—C9—O2	118.1 (2)	O3—C22—H22C	109.5
C11—C10—C9	119.5 (3)	H22A—C22—H22C	109.5
C11—C10—H10	120.3	H22B—C22—H22C	109.5
C9—C10—H10	120.3	C8—N1—C5	125.9 (2)
C10—C11—C12	120.0 (3)	C15—N2—C14	117.6 (2)
C10—C11—H11	120.0	C15—N3—C16	130.4 (2)
C12—C11—H11	120.0	C15—N3—H3A	114.8
C13—C12—C11	120.6 (3)	C16—N3—H3A	114.8
C13—C12—H12	119.7	C2—O1—C1	118.4 (2)
C11—C12—H12	119.7	C8—O2—C9	118.8 (2)
C12—C13—C14	119.9 (3)	C19—O3—C22	118.5 (2)
			
O1—C2—C3—C4	−177.7 (3)	C17—C18—C19—O3	179.2 (3)
C7—C2—C3—C4	2.0 (4)	C17—C18—C19—C20	−0.6 (5)
C2—C3—C4—C5	1.3 (4)	O3—C19—C20—C21	−179.8 (3)
C3—C4—C5—C6	−3.4 (4)	C18—C19—C20—C21	0.0 (4)
C3—C4—C5—N1	177.9 (2)	C19—C20—C21—C16	0.5 (5)
C4—C5—C6—C7	2.3 (4)	C17—C16—C21—C20	−0.4 (4)
N1—C5—C6—C7	−179.3 (3)	N3—C16—C21—C20	−179.8 (3)
O1—C2—C7—C6	176.6 (3)	O2—C8—N1—C5	0.2 (4)
C3—C2—C7—C6	−3.2 (4)	C15—C8—N1—C5	178.3 (2)
C5—C6—C7—C2	1.0 (5)	C6—C5—N1—C8	25.8 (4)
C14—C9—C10—C11	0.6 (4)	C4—C5—N1—C8	−155.7 (3)
O2—C9—C10—C11	−178.2 (3)	N3—C15—N2—C14	−178.2 (2)
C9—C10—C11—C12	0.1 (4)	C8—C15—N2—C14	2.6 (3)
C10—C11—C12—C13	−0.4 (5)	C9—C14—N2—C15	1.1 (4)
C11—C12—C13—C14	−0.1 (4)	C13—C14—N2—C15	−179.9 (2)
C10—C9—C14—N2	177.9 (2)	N2—C15—N3—C16	3.1 (4)
O2—C9—C14—N2	−3.4 (4)	C8—C15—N3—C16	−177.7 (2)
C10—C9—C14—C13	−1.1 (4)	C21—C16—N3—C15	−171.6 (3)
O2—C9—C14—C13	177.7 (2)	C17—C16—N3—C15	9.0 (5)
C12—C13—C14—C9	0.9 (4)	C3—C2—O1—C1	−176.0 (3)
C12—C13—C14—N2	−178.1 (3)	C7—C2—O1—C1	4.3 (5)
N1—C8—C15—N2	177.6 (2)	N1—C8—O2—C9	−180.0 (2)
O2—C8—C15—N2	−4.3 (4)	C15—C8—O2—C9	2.0 (3)
N1—C8—C15—N3	−1.7 (3)	C14—C9—O2—C8	1.6 (4)
O2—C8—C15—N3	176.5 (2)	C10—C9—O2—C8	−179.6 (2)
C21—C16—C17—C18	−0.2 (4)	C18—C19—O3—C22	12.6 (5)
N3—C16—C17—C18	179.2 (3)	C20—C19—O3—C22	−167.6 (3)
C16—C17—C18—C19	0.7 (5)		

### Hydrogen-bond geometry (Å, °)

**Table:**

*D*—H···*A*	*D*—H	H···*A*	*D*···*A*	*D*—H···*A*
C7—H7···O3^i^	0.93	2.59	3.423 (3)	149

Symmetry codes: (i) *x*−1/2, −*y*+3/2, *z*+1/2.

## References

[bb8] Asgari, D., Mehrdad, M., Ghanbari, M., Jadidi, K., Behzad, S. K. & Khavasi, H. R. (2011). *Acta Cryst.* E**67** Submitted [BT5429]

[bb1] Azizian, J., Mehrdad, M., Jadidi, K. & Sarrafi, Y. (2000). *Tetrahedron Lett.* **41**, 5265–5268.

[bb7] Farrugia, L. J. (1997). *J. Appl. Cryst.* **30**, 565.

[bb2] Farrugia, L. J. (1999). *J. Appl. Cryst.* **32**, 837–838.

[bb3] Jadidi, K., Ghahremanzadeh, R., Mehrdad, M., Ghanbari, M. & Arvin-Nezhad, H. (2008). *Monatsh. Chem.* **139**, 277–280.

[bb4] Mehrdad, M., Ghanbari, M., Jadidi, K., Asgari, D. & Khavasi, H. R. (2011). In preparation.

[bb5] Sheldrick, G. M. (2008). *Acta Cryst.* A**64**, 112–122. 10.1107/S0108767307043930 18156677

[bb6] Stoe & Cie (2005). *X-AREA* Stoe & Cie, Darmstadt, Germany.

